# Design Optimization of the Mechanics of a Metamaterial-Based Prosthetic Foot

**DOI:** 10.3390/ma18010096

**Published:** 2024-12-29

**Authors:** Agata Mrozek-Czajkowska, Tomasz Stręk

**Affiliations:** Faculty of Mechanical Engineering, Poznan University of Technology, Piotrowo 3 Street, 61-138 Poznan, Poland; agata.mrozek-czajkowska@put.poznan.pl

**Keywords:** foot prosthesis, metamaterials, auxetics, optimization, virus optimization algorithm

## Abstract

This paper is dedicated to the analysis of a foot prosthesis optimization process, with a particular focus on the application of optimization algorithms and unconventional materials, such as auxetic materials. The study aims to enhance prosthesis performance by minimizing the difference between the ground reaction force generated by the prosthetic foot and that of a natural limb. In the initial part of the study, the basic topics concerning the parameterization of the foot prosthesis geometry and the preparation of a finite element model for human gait are discussed. In the subsequent part of the study, the focus is on the optimization process, in which algorithms were applied to adjust the prosthesis structure to the patient’s individual needs. The optimization process utilized a finite element method gait model. After validating the FEM, an algorithm generating the prosthesis geometry based on the given parameters was developed. These parameters were optimized using the VOA, comparing FEM gait model data on vertical ground reaction force with experimental results. The results of the foot prosthesis optimization are presented through a comparison of different structural models. The study also demonstrates the application of auxetic materials, which, due to their unique mechanical properties, can enhance foot prosthesis efficiency. Simulations were performed using multi-material topology optimization. The results obtained for different gait phases were compared.

## 1. Introduction

Foot prostheses enhance comfort and effectiveness, allowing individuals with lower limb amputations to restore the ability to move independently. A prosthetist’s primary task is to adjust prosthetic components to achieve an optimal balance between functionality and cost. Decisions regarding the selection of specific components are also influenced by users’ personal preferences—from those requiring safety and mobility recovery for everyday tasks to athletes expecting maximum comfort and efficiency from their prosthetic devices during sports.

The stiffness of the prosthesis significantly influences the biomechanical gait parameters. A higher flexibility results not only in an increased range of motion, it also enhances generated power and energy return. Moreover, high stiffness is necessary to maximize stability during standing. The design of the prosthesis must therefore consider the compromise between the need to support the appropriate body weight and maximizing energy return [1,2,3,4,5,6,7,8,9,10,11,12,13,14].

Lower limb amputation results in significant challenges to daily mobility. The design and various mechanical properties of the foot prosthesis affect the gait pattern and user comfort. During walking, individuals with lower limb amputations experience increased ground reaction forces on the healthy limb due to the limited rebound of the prosthetic foot. This phenomenon causes rapid weight transfer to the healthy limb and shorter steps with the prosthetic limb. Consequently, the resulting gait asymmetry leads to joint pain and degradation, ultimately causing osteoarthritis in the healthy limbs of individuals with amputations [15,16].

A primary objective in foot prosthesis optimization is achieving a gait pattern similar to the pattern of able-bodied individuals. Early efforts focused on replicating the physiological geometry of foot roll-over during walking [17]. Nonetheless, roll-over geometry is measured exclusively within the ankle–knee reference system and provides no data regarding the orientation of this system relative to the global reference system. As a result, two prostheses may share identical roll-over geometries while giving significantly divergent lower limb kinematics. Thus, roll-over geometry alone is insufficient for optimizing the design of foot prostheses [18,19].

Another critical parameter in the optimization of foot prostheses is lower limb trajectory error (LLTE). LLTE defines the difference between the trajectory predicted for a specific foot prosthesis, determined by the deformed shape of the foot under typical ground reaction forces, and the target physiological trajectory of the lower limb derived from established gait datasets [18,19,20,21]. Studies indicate that the LLTE approach can be effectively employed to design personalized, high-performance prostheses using cost-effective materials.

Individuals with unilateral below-knee amputations exhibit an increased metabolic cost and gait asymmetry compared to non-disabled individuals. In response, Fey developed an optimization process for the stiffness of the energy-storing and returning (ESAR) foot prosthesis, utilizing dynamic gait simulations [22]. Through dynamic optimization, muscle activation patterns and foot stiffness profiles were identified that produced simulations aligning with experimental gait data for individuals with amputations, while minimizing both metabolic cost and internal knee contact forces on the healthy limb. The analyses revealed that modifying the nominal stiffness distribution of the foot prosthesis—specifically by increasing the stiffness of the toes and midfoot while reducing the stiffness of the ankle and heel—enhanced the ESAR foot’s performance. These adjustments resulted in reduced loading on the healthy knee during the early to mid-stance phase and decreased metabolic cost.

Topology optimization is applied to improve foot prosthesis designs by defining the spatial distribution of material within a specific area, meeting specified conditions, and minimizing a defined objective function [23]. A key objective of topology optimization in lower limb prostheses is to minimize mass, which significantly affects the prosthesis’s functionality [24,25,26,27]. One example of input data for topology optimization is pressure distribution data obtained from a baropodometric mat. The pressure distribution during the gait cycle provides an opportunity to customize prostheses, thereby reducing motion asymmetry between the healthy limb and the residual limb [28].

Another approach to perform the optimization process is to apply heuristic algorithms inspired by nature. In 2016, Liang and Cuevas Juarez introduced a novel metaheuristic approach known as the Virus Optimization Algorithm (VOA) [29]. They analyzed its performance by solving eight benchmark functions. Since its development, the algorithm has found applications in various fields. The VOA was applied to detect potential damage to a wind turbine blade by application of the finite element method [30], to forecast the spread of COVID-19 in the USA based on population density and climate parameters [31], or to predict mortality in Thailand [32]. Grabski and Mrozek determined the elastoplastic properties of rods based on torsion test data using the VOA in conjunction with the method of fundamental solutions and radial basis functions [33]. Additionally, the same authors optimized the parameters of a four-bar mechanism to ensure it accurately followed a prescribed trajectory [34].

Metamaterials offer novel opportunities to enhance prosthetic design by enabling the development of customizable structural properties that closely replicate natural biomechanics, thereby improving user comfort and functionality. The term “metamaterial” refers to a synthetic composite material engineered to exhibit unconventional electromagnetic, acoustic, or mechanical properties, such as a negative refractive index or negative magnetic permeability. These unique properties arise from the material’s structure at a scale larger than the molecular level [35].

Metamaterials are increasingly being applied in various engineering fields due to their unique mechanical and functional properties. Recent advancements have demonstrated their effectiveness in high-performance applications such as thermal management, optical systems, and advanced material design [36,37,38].

Among the various types of metamaterials, auxetic metamaterials are particularly significant. Auxetic metamaterials are distinguished by their negative Poisson’s ratio, which represents a negative relationship between transverse and longitudinal strain [35].

When a conventional material is compressed, its cross-section perpendicular to the compression direction expands. Conversely, during stretching, a reduction in cross-sectional dimensions is observed. In auxetic metamaterials, however, the behavior is reversed: a decrease in cross-sectional area occurs during compression, while an increase is observed during stretching [35,39,40,41,42]. The characteristic mechanical properties of auxetic metamaterials, including their unconventional deformation behavior, high energy absorption, and impact resistance, have stimulated intensive research into their potential use in designing personal protective equipment, such as helmets and ballistic barriers [43,44]. Furthermore, the growing interest in the medical applications of these materials has attracted considerable attention from researchers.

A fundamental characteristic of auxetic metamaterials is their negative Poisson’s ratio, which enables a more precise adaptation to anatomical shapes [45]. Prosthetic sockets are the component of a prosthesis that requires the highest degree of customization, as they are responsible for transferring forces between the prosthesis and the patient’s limb. Incorporating metamaterials can optimize prosthetic equipment by enhancing socket comfort and reducing pressure experienced by the patient [46,47]. The potential to improve patient comfort and safety through the use of auxetic metamaterials was also highlighted by Kowalczyk [48]. Additionally, studies [49,50] have investigated the incorporation of auxetic structures around the toes and heel to effectively mimic the functions of the human foot. Duncan and co-authors [41] presented a review of auxetic materials for sports applications.

The main focus of this study is optimizing geometric and material design parameters to enhance the mechanical performance and biomechanical functionality of foot prostheses. This research aims to address a key challenge encountered by individuals with lower limb amputations: increased ground reaction forces on the healthy limb. This imbalance leads to rapid weight transfer, shorter steps on the prosthetic limb, and gait asymmetry, which can cause joint pain, joint degeneration, and eventually osteoarthritis in the healthy limb.

By addressing this issue, the study aims to enhance prosthetic designs that reduce asymmetry. These improvements could mitigate the long-term biomechanical complications experienced by individuals using prosthetic devices.

This study explores the optimization of foot prostheses by combining advanced computational methods and innovative materials. The study begins with an examination of the parameterization process for prosthesis geometry and the development of a finite element model (FEM) to simulate human gait. Subsequently, an optimization process is applied, using algorithms such as the Virus Optimization Algorithm (VOA) to adjust the prosthesis design based on individual patient needs. Additionally, the potential of auxetic materials to improve prosthetic efficiency was investigated. The study applied multi-material topology optimization to simulate various gait phases, presenting a comprehensive analysis of structural models and their performance.

## 2. Materials and Methods

### 2.1. Geometry

The development of the prosthesis optimization process started with the creation of a parametric model of the prosthesis, allowing modification of crucial elements critical to gait performance. This approach facilitates the simulation of different sets of parameters. Their impact on the obtained vertical ground reaction force is analyzed.

**Figure 1:**
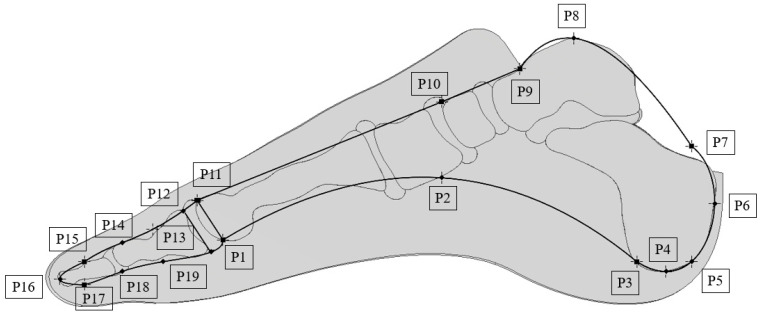
A basic model of the internal structure of the prosthesis.

The prosthesis model was designed based on the dimensions of the anatomical foot, with nineteen key points defined to represent its structure (Figure 1). Three main components were identified in the prosthesis design: a segment responsible for the heel area, a component covering the metatarsophalangeal joint, and a section designed for the toes. Each component performs a distinct function, working in accordance to replicate the natural movements of the foot as accurately as possible.

**Figure 2:**
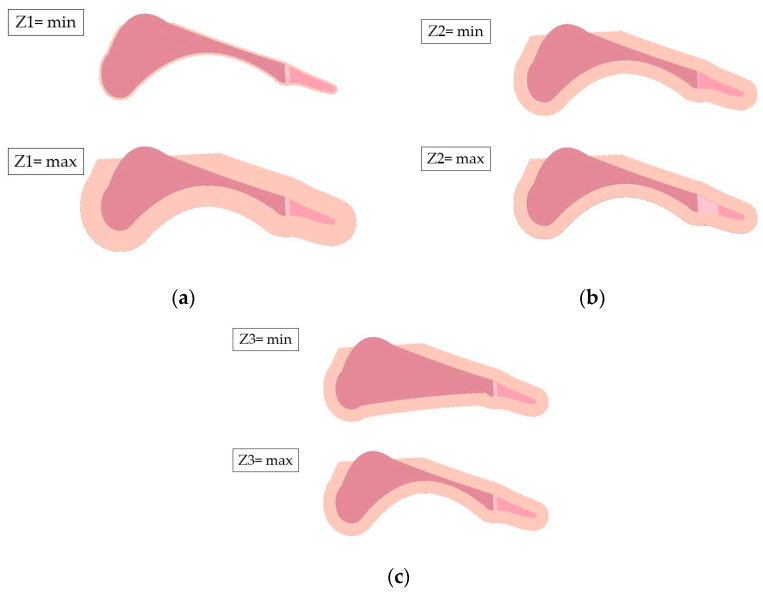
Geometrical parameters defined for optimization: (**a**) parameter Z1; (**b**) parameter Z2; (**c**) parameter Z3.

During the optimization process, the influence of three geometric parameters on the vertical ground reaction force was analyzed. The first parameter, Z1, specifies the offset of the outer edge of the sketch relative to the keel geometry (Figure 2a). The second parameter, Z2, determines the thickness of the metatarsophalangeal joint (Figure 2b). Furthermore, two longitudinal arches are present in the anatomical structure of the foot. The impact of the radius of the medial longitudinal arch, designated as Z3, was also examined. This arch was defined between points P1 and P3, with the radius value adjusted by altering the height of point P2 relative to point P1 (Figure 2c).

The developed geometry was applied to the gait finite element model. To achieve this, the geometry needed to be properly oriented in space to ensure that the angle of the prosthesis relative to the ground was set to 27.5°.

### 2.2. Finite Element Model of Gait

The creation of the finite element model required conducting physical tests to obtain input data for the model. Based on data obtained from experimental gait tests recorded using the BTS Smart motion capture system, the boundary conditions, applied loads, and initial conditions were determined. These conditions were validated through a comparison between the finite element analysis (FEA) of the anatomical foot and the experimental data.

**Figure 3:**
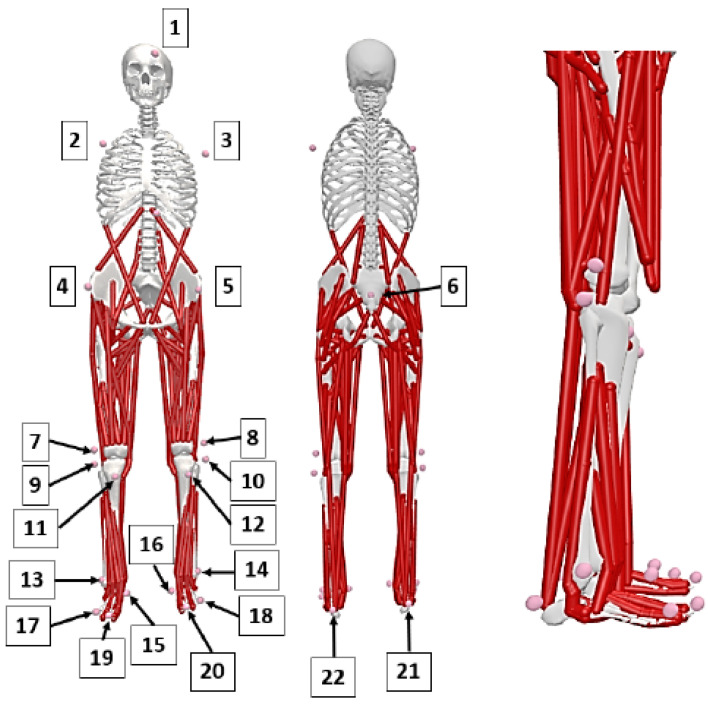
Applied marker set: 1—Head, 2—R. Acromium, 3—L. Acromium, 4—RASI, 5—LASI, 6—Sacrum, 7—RKNE, 8—LKNE, 9—RHFB, 10—LHFB, 11—RTUB, 12—LTUB, 13—RANK, 14—LANK, 15—RP1M, 16—LP1M, 17—RP5M, 18—LP5M, 19—RTOE, 20—LTOE, 21—RHEE, 22—LHEE.

The experimental validation involved capturing both kinematic and kinetic data related to human gait. The kinematic data were collected using a BTS Smart motion capture system equipped with 6 cameras. A total of 22 reflective markers were placed on key anatomical landmarks of the participant’s body (Figure 3). Marker movements were recorded at a frequency of 250 Hz, enabling the precise tracking of gait motion.

Ground reaction forces were measured using two AMTI force plates embedded in a specially prepared walkway. These force plates captured forces in three directions at a sampling rate of 500 Hz. During each trial, only one foot made contact with a force plate at a time, ensuring accurate measurement of individual foot–ground interaction forces.

The experimental protocol involved the participant walking along the prepared walkway while the motion capture system and force plates simultaneously recorded kinematic and kinetic data. A total of 15 valid trials were collected to obtain averaged results, enhancing the reliability of the data used for FEM validation.

**Figure 4:**
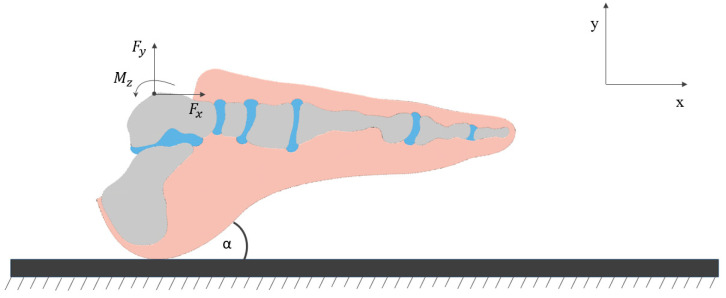
Defined boundary conditions and applied loads.

Initial conditions, encompassing the horizontal and vertical velocities of the ankle joint, the angle of the foot relative to the ground, and the angular velocity, were defined based on the recorded kinematic data at the moment of heel strike on the fixed ground. The loading was applied in the form of forces (comprising vertical and horizontal components) and the net muscle moment for the ankle joint throughout the entire stance phase. These loading conditions, calculated using the inverse dynamics method [51,52] and based on the measured segmental motions and ground reaction forces, were first validated through an analysis of the anatomical foot and then applied to the prosthetic gait model. The applied loading is illustrated in Figure 4.

The study primarily focuses on presenting boundary conditions in the sagittal plane to reduce computational costs and simplify the analysis by treating the model as a 2D problem. Nevertheless, due to the limitations of the FEBio software (version 2.6.0), which does not natively support 2D analysis, a single layer of 3D elements was incorporated, and constraints were applied along the z-axis. This method allowed the problem to be effectively analyzed while compliance with the software’s requirements.

**Figure 5:**
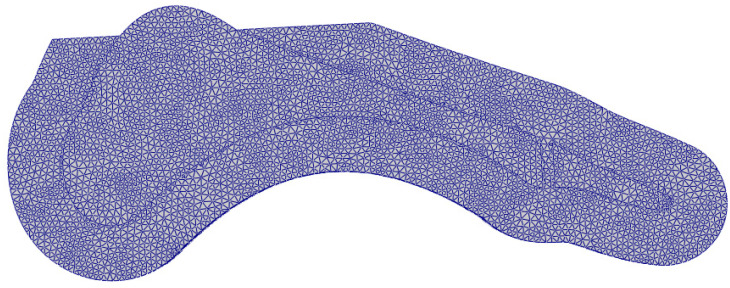
Applied finite element mesh.

The geometry of the ground was fixed, while a sliding-type contact was defined between the outer shell and the ground. The discretization of the resulting geometry was performed using two types of elements: C3D4 and C3D8. The defined target element length for mesh was equal to 2 mm. The finite element mesh applied during optimization process used an average of 28,760 elements (Figure 5). The keel elements were modeled as rigid bodies. The prosthesis shell and the part imitating the metatarsophalangeal joint were modeled using a neo-Hookean hyperelastic material model (Table 1). FEAs were performed in FEBio software [53].

### 2.3. Virus Optimization Algorithm

The objective of the applied optimization procedure was to minimize the difference between the ground reaction force obtained from FEM simulations of prosthetic gait and the experimentally measured value for a physiological foot. Various geometric parameters were considered during the simulations. This approach aimed to increase the similarity between the prosthesis’ behavior and that of a natural foot. The vertical component of the ground reaction force derived from the simulations enables the calculation of the objective function value (E), defined as

(1) $$E=\frac{1}{N}\sum_{i=1}^{N}\frac{\left|F_{FEA}\left(i\right)-F_{exp}\left(i\right)\right|}{F_{exp}\left(i\right)},$$

where N represents the number of samples, and F_exp_ and F_FEA_ correspond to the experimentally measured force and the force obtained from the FEM analysis, respectively.

The input for the objective function is a vector containing a set of optimized parameters (Z1, Z2, Z3). Three geometric parameters were selected and analyzed within the ranges presented in Table 2. Based on these parameters, the geometry is generated, and an input file for the FEBio environment (*.feb) is automatically created. In the first step, a Design of Experiments (DoE) analysis was conducted, followed by the application of the VOA optimization algorithm to simultaneously optimize the values of all defined parameters.

The VOA, which mimics the behavior of a virus when attacking a host cell, is used in continuous optimization. The VOA distinguishes between three main steps: initialization, replication, and maintenance.

**Figure 6:**
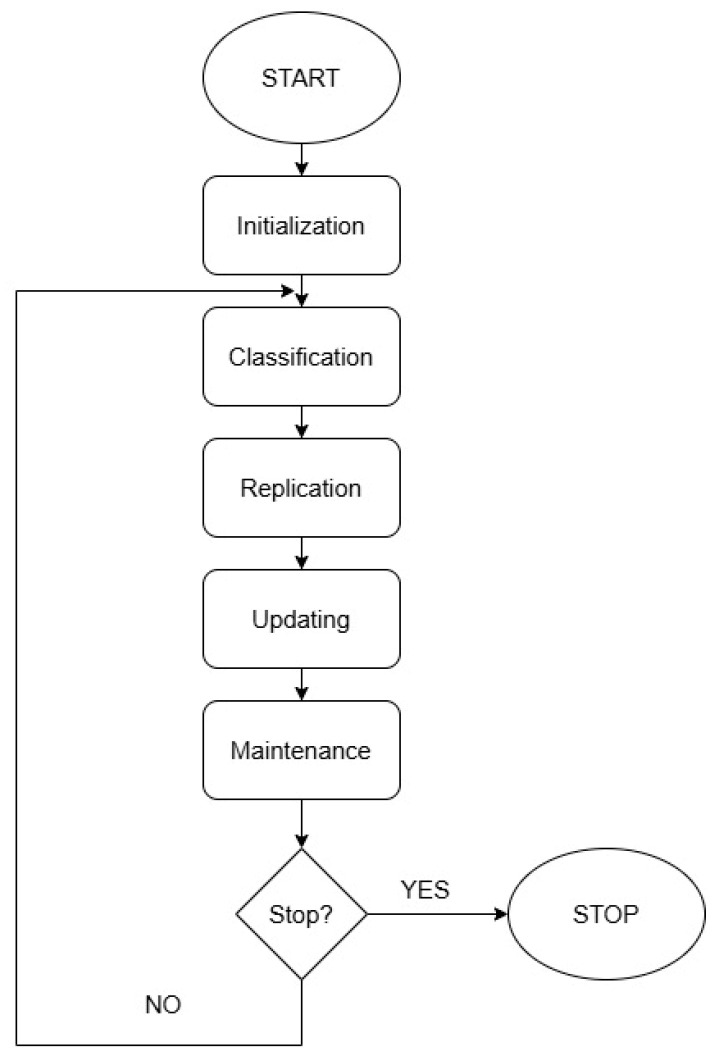
Pseudocode of the VOA.

The first population of viruses is created in the initialization process (Figure 6). The following step is the random generation of the initial viruses. Viruses are classified as either strong (SVs) or common (CVs). During the replication procedure, a certain number of new viruses (NVs) are created from both strong and common members of the population. The newly generated viruses are calculated according to the following equations:

(2) $${NV}_{kl}={SV}_{kl}\pm \frac{rand()}{intensity}{SV}_{kl},$$

(3) $${NV}_{kl}={CV}_{kl}\pm rand(){CV}_{kl},$$

where the indices k and l denote the k-th member of the population in the l-th dimension, while rand represents a uniformly distributed random number between 0 and 1. A parameter called intensity determines the rate at which new strong viruses are generated. The third stage, maintenance, occurs as a result of interactions between the defined viruses and host cells. The population size decreases if the number of viruses present in the host cell exceeds a certain number.

The tool for evaluating the population is the average value of the objective function. Viruses that give the worst results in terms of average values of the objective function are removed. The number of population members killed (KMs) is determined according to the following equation:

(4) KM=rand(0,NP−NSV)

where NP is the population size, which indicates the current number of viruses in the host cell, and NSV is the number of strong viruses.

The algorithm has five parameters whose values have to be defined. These include the number of initial solutions (NIS), the number of strong viruses (NSV), the growth rate of new solutions from strong and common viruses (GRSV and GRCV), and the stopping criterion (maximum number of replications—MNR) [29,33,34]. For the VOA, the effect of individual algorithm parameters on the obtained value of the objective function was investigated. The ranges of the variables tested are shown in Table 3. The initial values were chosen based on preliminary testing, with NIS set to 1 and MNR to 3, providing reasonable initial results. The maximum values of NIS and MNR were set to 5 and 25, respectively, to ensure the best optimization performance while keeping computation time manageable.

### 2.4. Multi-Material Topology Optimization

Topology optimization is a widely utilized methodology in engineering. Nevertheless, its application is mainly limited to structures consisting of a single type of material. The concept of multi-material topological optimization facilitates the advanced design of prosthetic devices through the integration of diverse materials within a unified structure. The discussion is grounded in an optimization algorithm originally developed within the MATLAB environment, as detailed in [54], which has been augmented with functionalities to support multi-material optimization, as described in [55]. The multi-material topology optimization algorithm used in this study has been fully described and published in scientific papers [54,55], where it is available for reference and further application.

The algorithm used during topology optimization is based on an approach in which each element is assigned a density value that determines material properties, in particular Young’s modulus. The goal of the optimization is to find the optimal distribution of material in the domain to minimize compliance (increase stiffness) while satisfying the volume constraint.

The density x_e_ of each element e is used to interpolate the Young’s modulus (E_e_). The interpolation uses a modified Solid Isotropic Material with Penalization (SIMP) method:

(5) $$E_{e}\left(x_{e}\right)=E_{min}+x_{e}^{p}\left(E_{0}-E_{min}\right),\;\;x_{e}\in \left[0,1\right]$$

where E_0_ is the stiffness of the material, E_min_ is the stiffness with a small value assigned to void areas to prevent the occurrence of singularities in the stiffness matrix, and the variable p denotes the penalty factor [55]. The objective function is to minimize the compliance (c), defined as:

(6) $$c\left(x\right)=U^{T}KU=\sum_{e=1}^{N}E_{e}(x_{e})u_{e}^{T}k_{0}u_{e},$$

where K is the global stiffness matrix, U is the global displacement vector, F is the global force vector, u_e_ is the element displacement vector, k_0_ is the element stiffness matrix for an element with unit Young’s modulus, x is the vector of design variables, and N is the number of elements used to discretize the design domain [54]. The algorithm has been extended to perform a multi-material optimization process.

The design space is divided into finite elements, which are assigned a density x_e_ to determine the material distribution in the topology optimization of continuous structures. For a single-material problem, only one design variable is used, while when multiple materials are considered in topology optimization, the number of design variables for multiple materials is equal to the number of materials multiplied by the number of elements, which can be expressed as:

(7) $$x=\left[\begin{array}{ccccc} x_{1}^{1} & x_{2}^{1} & \cdots & x_{NM-1}^{1} & x_{NM}^{1}\\ x_{1}^{2} & x_{2}^{2} & \cdots & x_{NM-1}^{2} & x_{NM}^{2}\\ \vdots & \vdots & & \vdots & \vdots \\ x_{1}^{NE-1} & x_{2}^{NE-1} & \cdots & x_{NM-1}^{NE-1} & x_{NM}^{NE-1}\\ x_{1}^{NE} & x_{2}^{NE} & \cdots & x_{NM-1}^{NE} & x_{NM}^{NE} \end{array}\right]$$

where NE is the number of finite elements and NM is the number of materials [56].

**Figure 7:**
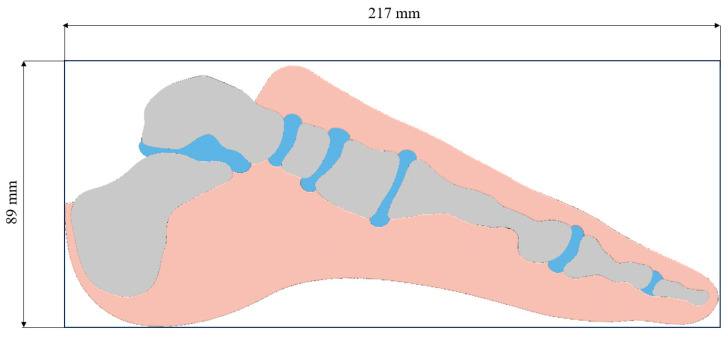
Defined design space.

The analysis of various load cases provides insight into the influence of different forces acting on the prosthesis and their impact on the resulting material distribution within the design space. The proposed design space is defined as a rectangular domain, with its dimensions determined based on the anatomical dimensions of the foot (Figure 7).

**Figure 8:**
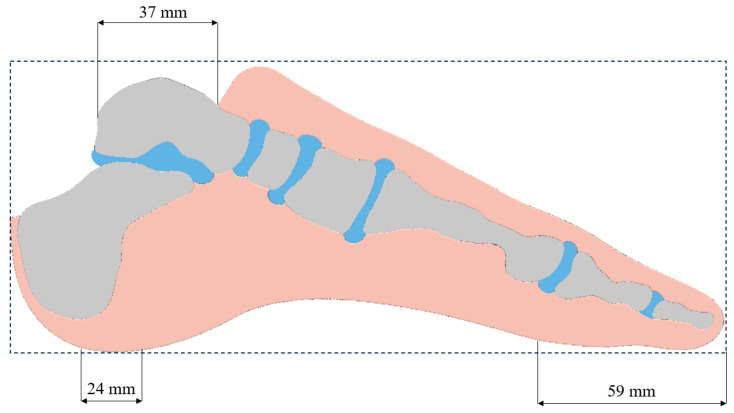
Visualization of regions of defined boundary conditions.

The optimization process considered three load cases according to the most essential phases of gait. The first involved heel strike on the ground, where the unit force was applied only at the lower edge of the design space, over a width of 24 mm. The second case involved a load limited to an area covering part of the toe. The third load case represented the middle support phase. The force was distributed over both previously described areas. In all three cases, the area of restraint remained the same—the upper surface, corresponding to the width of the talus in the sagittal plane (Figure 8).

Each load case was evaluated separately during the initial phase of the analysis. Three distinct materials were considered, with properties listed in Table 4. This approach facilitated a comparative assessment of the structural behavior depending on the materials employed. The multi-material optimization identified the distributions of two materials: auxetic and conventional (non-auxetic). For the auxetic material, the Young’s modulus was assigned as specified in Table 4, while the Poisson’s ratio was assumed to be the negative value of the coefficient defined for the original material (Material 1—auxetic; Material 2—conventional material). In the second procedure, the algorithm allowed eleven different values of Poisson’s coefficients to be considered for a given material. The material number and corresponding Poisson’s ratio are shown in Table 5.

## 3. Results

### 3.1. Foot Prosthesis Parametric Optimization

#### 3.1.1. Design of Experiments Analysis

The first stage of optimization involved conducting DoE analysis for three selected geometric parameters. The objective of this analysis was to investigate the influence of individual parameters on the value of the objective function. Each parameter was analyzed independently. This approach enabled a precise assessment of its impact on the vertical component of the ground reaction force.

**Figure 9:**
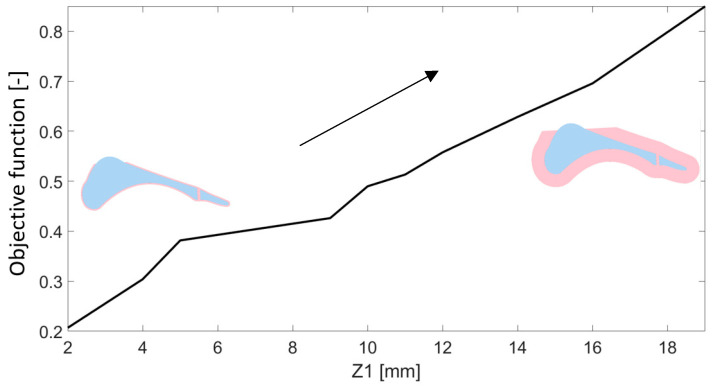
Influence of parameter Z1 on the obtained value of the objective function.

The first parameter, Z1, determines the offset of the outer edge of the sketch relative to the keel geometry. This offset defines the thickness of the shell surrounding the prosthesis core. This parameter was evaluated in the range of 2 to 19 mm. Figure 9 shows the correlation between the calculated value of the objective function and the thickness of the prosthesis shell. The smallest relative error observed between experimental data and simulation results corresponded to a parameter value of 2 mm. As the thickness of the shell increased, a proportional increase in the objective function was observed, with values ranging from 0.21 to 0.85.

**Figure 10:**
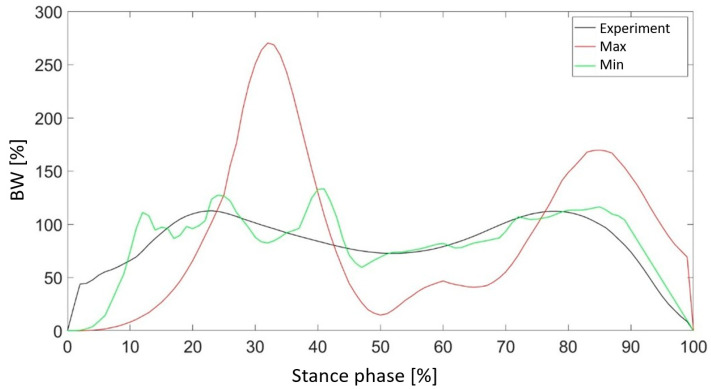
Comparison of the vertical component of the ground reaction force for the value of the parameter Z1 with the maximum and minimum values of the objective function.

Figure 10 presents a comparison of the vertical component of the ground reaction force for cases characterized by the highest and lowest values of the objective function. Additionally, the results captured during experiments conducted with a physiological foot are included. Reducing the thickness of the outer shell allows for a more accurate representation of the vertical component of the ground reaction force, particularly in phases where local force maxima occur. Nevertheless, this case exhibited a higher frequency of local fluctuations in the force values. Conversely, for the maximum shell thickness, a shift in the occurrence of the force maximum was observed relative to the experimental results.

**Figure 11:**
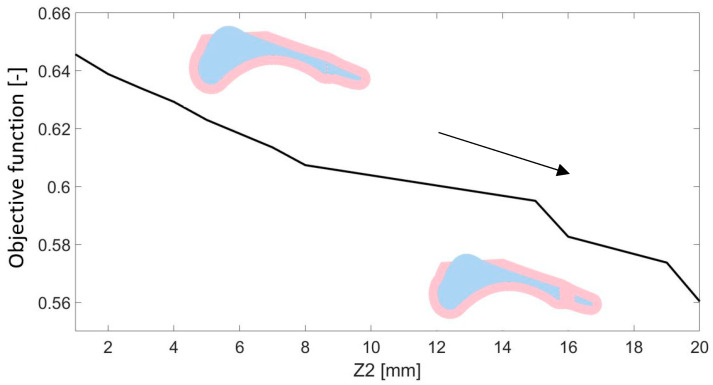
The influence of parameter Z2 on the obtained value of the objective function.

**Figure 12:**
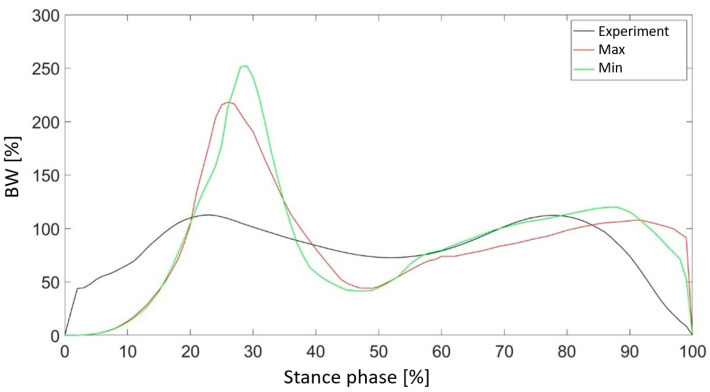
Comparison of the vertical component of the ground reaction force for the value of the parameter Z2 with the maximum and minimum values of the objective function.

The Z2 parameter, which determines the thickness of the metatarsophalangeal joint, assumed values ranging from 1 to 20 mm. In this case, a negative correlation was observed between the value of the parameter and the accuracy of reproducing the value of the vertical component of the ground reaction force (Figure 11). As the area of the hyperelastic component within the metatarsophalangeal joint became larger, the value of the objective function decreased. It was also noted that although increasing the parameter had a negative effect on the obtained mapping in the phase associated with heel-strike contact with the ground, during the heel-off phase the graph from the simulation overlapped with the values of the force measured for the patient’s foot (Figure 12).

**Figure 13:**
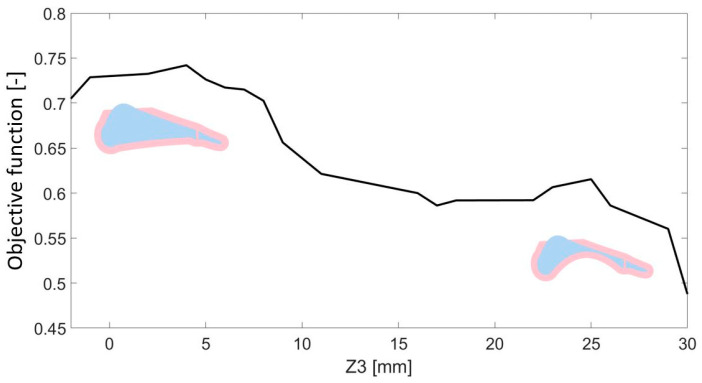
Influence of parameter Z3 on the obtained value of the objective function.

**Figure 14:**
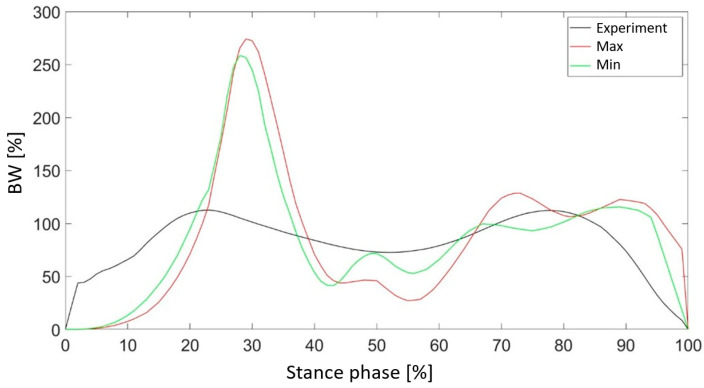
Comparison of the vertical component of the ground reaction force for the value of the parameter Z3 with the maximum and minimum values of the objective function.

The final parameter, Z3, represents the difference between the heights of points P1 and P2. This value defines the medial longitudinal arch radius. This parameter was analyzed in the range from −2–30 mm. For the following parameter, a negative correlation was also observed, although the relationship was not as strongly linear as in the case of the Z2 parameter. The value of the objective function in this case reached values in the range of 0.49 to 0.71 (Figure 13). Although Z3 did not significantly improve vertical ground reaction force representation during the heel-strike phase, it substantially improved the curve fit during the propulsion phase (Figure 14).

#### 3.1.2. Virus Optimization Algorithm

The previous chapter compared the effects of three geometric parameters of the prosthesis in a DoE analysis, with each parameter studied separately. This chapter discusses the results obtained using the VOA, which optimizes all three parameters simultaneously. This approach allows for a more comprehensive analysis of their interdependencies and impact on simulation results.

The first step of optimization using the VOA was to calibrate the parameters of the algorithm. The values of the parameters were changed in the range shown in Table 3. The MNR and NIS parameters had the greatest impact on the obtained objective function.

**Figure 15:**
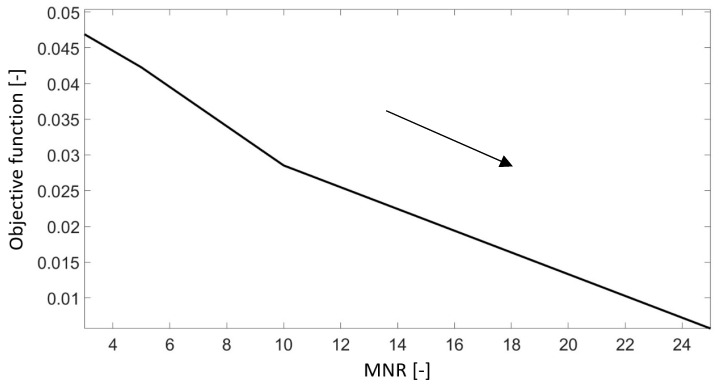
The influence of the MNR parameter on the obtained value of the objective function.

The value of the MNR parameter was studied in the range from 3 to 25 (Figure 15). Increasing the maximum number of replications results in a significant minimization of the value of the objective function—until the value equaled 0.0057. A similar value of the objective function (0.0055) was obtained when increasing the NIS parameter. The initial value of the objective function (for NIS = 1) equaled 0.03. The obtained solutions are shown in Table 6.

**Figure 16:**
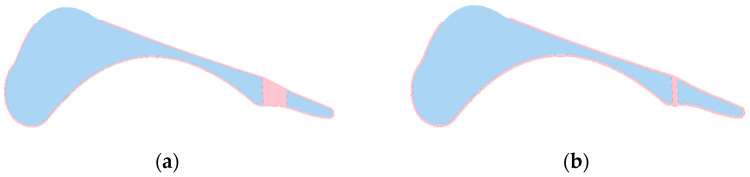
Geometry visualization for the selected objective function: (**a**) 0.0057 [-]; (**b**) 0.0055 [-].

Table 6 presents the obtained values for the geometric parameters of the structure. The value of the Z1 parameter is around 1 mm, which confirmed the results obtained in the DoE analysis. Similarly, for the Z3 parameter—the higher its value, the lower the value of the objective function. Nevertheless, it was observed that the values of the Z2 parameter for the selected solutions differ significantly. Visualization of the structures with the parameters presented in Table 6 is shown in Figure 16.

**Figure 17:**
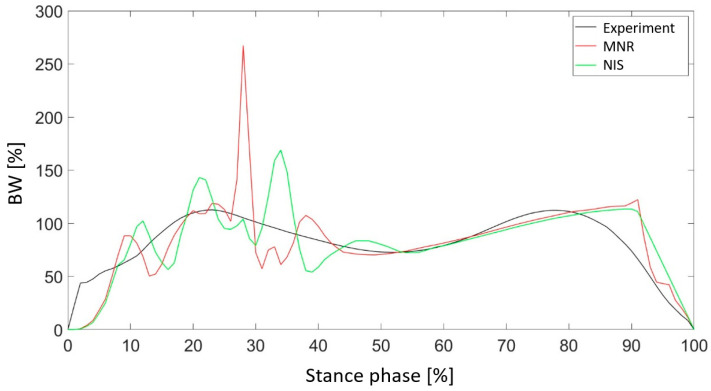
The vertical component of the ground reaction force for selected geometries characterized by the minimum value of the objective function.

Figure 17 shows the vertical ground reaction force for two sets of parameters characterized by the lowest value of the objective function. The application of the VOA allowed for a significant improvement in the reproduction of the curve in both the heel-strike and propulsion phases. Due to the small thickness of the outer shell, characteristic oscillations appear, as in Figure 10. This was observed only during heel contact with the ground. The small thickness of the material absorbing the impact of the foot on the ground is responsible for this phenomenon.

The optimization algorithm analyzed the influence of all three parameters simultaneously. This approach effectively reduced the value of the objective function. In the conducted tests, the value of the objective function, obtained by the VOA, was 97.38% lower compared to the smallest value obtained during the DoE analysis. However, the algorithm, despite its effectiveness in optimization, has a significant impact on increasing the calculation time. Table 7 compares computation times for minimal and maximum parameter values. The results show that the advanced optimization approach substantially increased computational cost.

The computational cost of the optimization process was evaluated by analyzing the impact of the maximum number of replications (MNR) and the number of initial solutions (NIS) on both the objective function value and processing time. These parameters play an essential role in determining the compromise between optimization accuracy and computational efficiency.

When the MNR parameter was set to its minimum value of 3, the objective function value reached 0.0469, with a corresponding computation time of 8623 s. Increasing MNR to its maximum value of 25 significantly reduced the objective function to 0.0057. Nevertheless, this improvement occurred at the cost of a substantially longer computation time of 34,134 s. This demonstrates that while increasing MNR enhances the optimization accuracy, it also leads to a considerable rise in processing time.

Similarly, when the NIS parameter was set to its minimum value of 1, the objective function value reached 0.0295, requiring 12,180 s of computation. Increasing NIS to 5 reduced the objective function value to 0.0055. The computation time increased to 82,151 s. This indicates that higher NIS values allow for a more precise search in the solution space but at a significant computational cost.

The results clearly highlight the correlation between optimization accuracy and computational efficiency. While higher values of MNR and NIS improve the quality of the optimization results by minimizing the objective function, they also cause a substantial increase in computation time. These findings underscore the importance of balancing these parameters to achieve optimal performance within acceptable computational limits. This balance is particularly relevant for real-time or large-scale optimization tasks where processing time may be a critical factor.

### 3.2. Multi-Material Topology Optimization

The topology optimization process conducted enabled obtaining material distribution maps in the defined design space. During the first procedure, the distribution of two materials was analyzed—with conventional properties (Table 4) and the corresponding auxetic material.

**Figure 18:**
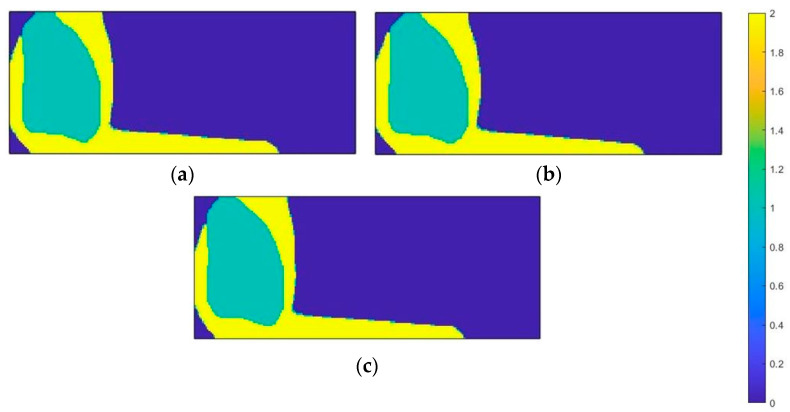
Material distribution map for the first load case—heel loading (material 1—auxetic material, material 2—conventional material): (**a**) onyx; (**b**) nylon 12; (**c**) steel.

Figure 18 shows the results obtained for the first loading case—the impact of the foot on the ground. For all three types of materials, the material distributions inside the design space were similar. The outer shell of the structure is made of conventional material—it surrounds the entire area of application of the force acting on the structure and most of the area of the nodes that were restrained. Therefore, the outer layer of material will tend to increase its width under compression. This material stabilizes the entire structure. The inner space, on the other hand, is filled with auxetic material.

The compression force applied to auxetic metamaterials causes deformation of the structure in the internal direction. This results in the compaction of the material, which allows for increased energy absorption, minimizing the risk of excessive stress on anatomical structures above the prosthesis. In addition, the distribution of the material in the area of fixation is not as uniform as in the case of the area of force application. The auxetic material is located at the top edge, defining the constraint area, which prevents stress concentration as the material reduces its volume and dissipates forces that could cause damage. In a material with a negative Poisson’s ratio, the internal energy resulting from stresses is dissipated over a larger area, leading to a reduction in the concentration of stresses. As a result, the vulnerability to damage is reduced and the material becomes more resistant to cracking. As a result, some of the fixation nodes are located in the area where the auxetic material is used.

**Figure 19:**
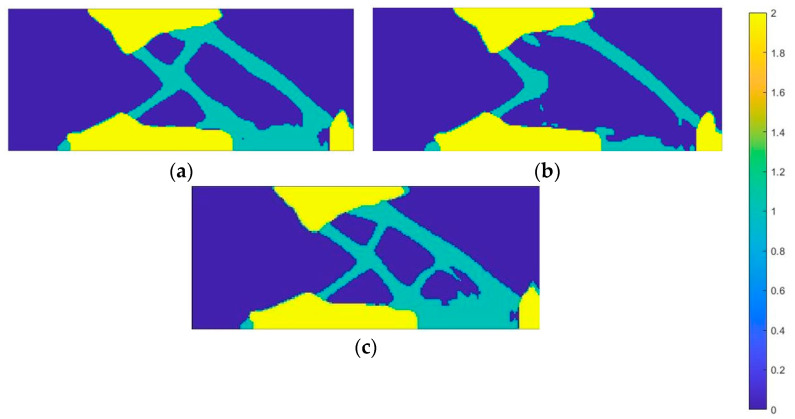
Material distribution map for the second load case—toe loading (material 1—auxetic, material 2—conventional material): (**a**) onyx; (**b**) nylon 12; (**c**) steel.

In the following load case, associated with the lifting off of the foot from the ground, the area where the force is applied, the toe region, is mostly assigned an auxetic material (Figure 19). The zone of distinction, on the other hand, is the tips of the toes—the proposed material for this area is a material with a positive Poisson’s ratio. In addition, an auxetic material was assigned between the loading region and the constraint, which promotes energy dissipation. The entire fixation area was made of conventional material, providing adequate stability.

**Figure 20:**
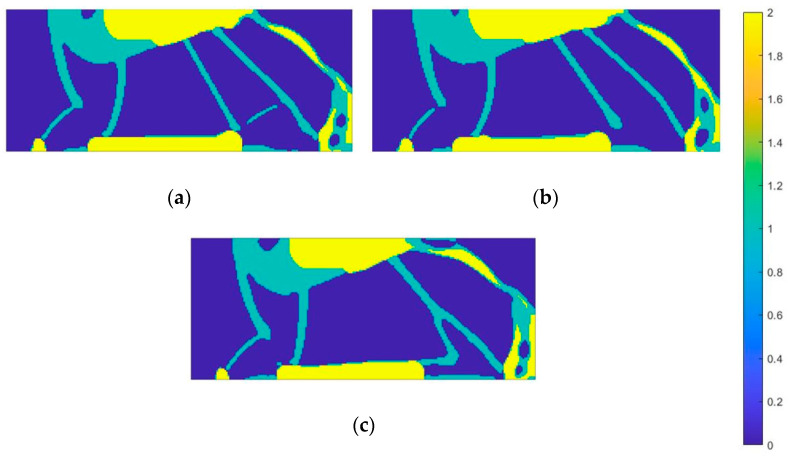
Material distribution map for the third load case—mid-stance phase (material 1—auxetic materials, material 2—conventional materials): (**a**) onyx; (**b**) nylon 12; (**c**) steel.

The last load case analyzed, corresponding to the mid-stance phase, results in a similar material distribution structure to the first case. The constraint area contains both auxetic and conventional materials, suggesting optimization of the restraint area for energy dissipation and stability. A distinguishing feature is the presence of only auxetic material between the load and constraint areas (Figure 20).

An additional layer of conventional material was applied to the lower edge of the structure. In previous cases, the auxetic material was surrounded by the conventional material. Nevertheless, in this case the situation is reversed—it is the conventional material that is surrounded by the auxetic material. In the toe area, both materials were used, with the toe span being reinforced with conventional material in the front, which provides balance and additional protection against excessive bending or damage. Comparing all three cases, it can be seen that the use of both materials in the constraint region is common in situations where the angle between the acting force and the constraint areas is around 90 degrees.

**Figure 21:**
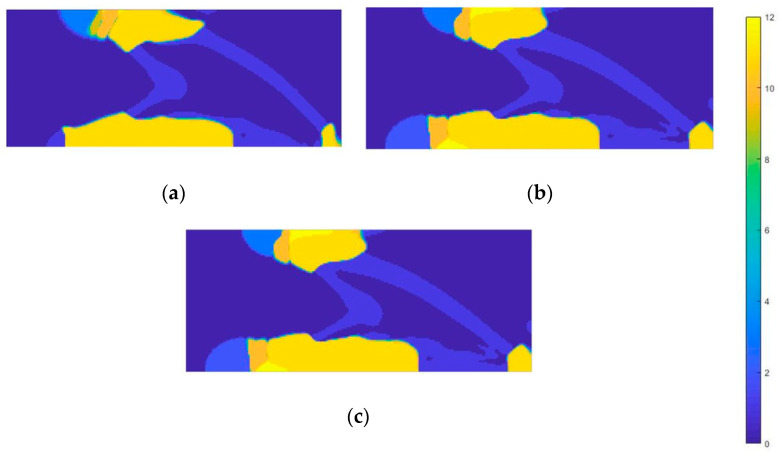
Material distribution map for the second load case—toe loading (materials 1–5: auxetic material, materials 6–11: conventional materials): (**a**) onyx; (**b**) nylon 12; (**c**) steel.

**Figure 22:**
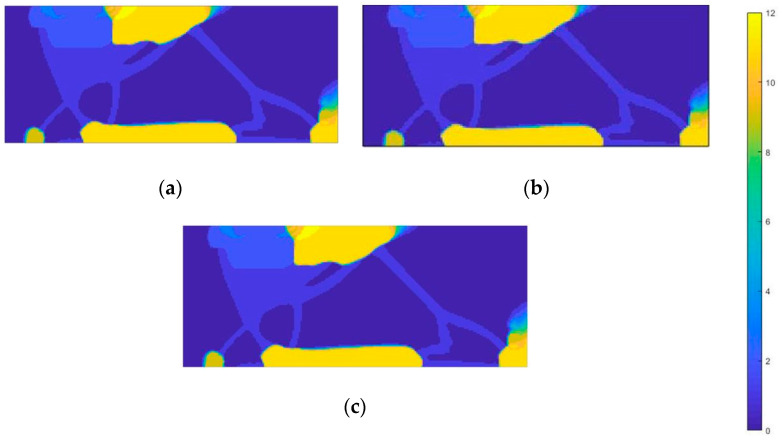
Material distribution map for the third load case—mid-stance phase (materials 1–5: auxetic materials, material 6–11: conventional materials): (**a**) onyx; (**b**) nylon 12; (**c**) steel.

The next procedure involved performing design space topology optimization for materials with Poisson’s ratio ranging from −0.5 to 0.5. Figure 21 and Figure 22 present the resulting material distribution maps.

Both auxetic and conventional materials are present in the constraint area. Auxetic materials near the restraint have absolute Poisson’s ratio values close to 0, around 0.2. Conventional materials in this region, on the other hand, have values in the upper range, around 0.4–0.5. The middle part of the structure is again made exclusively of auxetic materials, but in this case their ratio values are lower, in the range of −0.4 to −0.5.

## 4. Discussion

The presented study highlights a comprehensive approach to optimizing prosthetic designs by applying multi-parameter analysis and advanced optimization algorithms. The findings provide critical insights into the interdependencies between geometry and material properties, emphasizing the potential for enhanced prosthetic performance and stability.

This research investigated the impact of geometric parameters on the vertical component of the ground reaction force. The DoE analysis of geometric parameters demonstrated the significant influence of shell thickness (Z1), metatarsophalangeal joint thickness (Z2), and medial longitudinal arc radius (Z3) on the vertical component of the ground reaction force.

Comparing the values of the objective function, the Z1 parameter exhibited the strongest optimization potential. This parameter showed the largest changes in the value of the objective function, highlighting its key role in improving simulation results. The Z1 parameter influenced both the objective function values and the shape of the force curve. This effect distinguished it from other parameters with less noticeable impact. The thinner outer shell (parameter Z1) allowed for a more accurate representation of the ground reaction force curve, especially in the phases of local force maxima. Nevertheless, this design introduced higher frequencies of local force fluctuations.

On the other hand, the smallest reduction in the value of the objective function was observed when analyzing different values of the Z2 parameter. A negative correlation was noted, with increasing thickness reducing the value of the objective function. Increasing the Z2 parameter reduced the objective function value, indicating better load distribution and enhanced propulsion during the heel-off phase. Parameter Z3 showed a non-linear correlation between the arc radius and the objective function, making it exceptional among the parameters.

The Z3 parameter, defining the medial longitudinal arch radius, exhibited a non-linear relationship with the objective function. While its effect on GRF during heel-strike was limited, it slightly improved the curve fit during the propulsion phase, demonstrating its role in ensuring smoother force transfer.

The proposed optimization algorithm enables patient-specific customization of the foot prosthesis geometry, which is a critical component of a lower limb prosthetic system. By tailoring geometric parameters such as shell thickness (Z1), metatarsophalangeal joint thickness (Z2), and medial longitudinal arch radius (Z3), the algorithm ensures a more precise fit and functional adaptation to individual biomechanical needs.

The finite element analysis (FEA) simulations were conducted using an open-source software platform, ensuring accessibility and transparency in the modeling process. After completing the optimization process, the algorithm provides optimized geometric parameters, which can be easily entered into a custom script to generate a fully individualized prosthesis geometry.

These improvements directly impact prosthetic performance by reducing gait asymmetry and minimizing the risk of joint overload in the healthy limb. Additionally, the flexibility of the algorithm supports the design of various prosthetic models, making it applicable to a wide range of clinical scenarios.

The second stage of geometric parameter optimization involved the use of the VOA, which allows for a comprehensive analysis of the interdependencies between defined design parameters and their impact on simulation results. The implemented optimization algorithm, which simultaneously analyzed all defined geometric parameters, effectively minimized the value of the objective function. In the experiments conducted, the VOA reduced the value of the objective function by 97.38% compared to the lowest value obtained in the DoE analysis. The use of the VOA significantly improved the representation of the ground reaction force curve in both the heel-strike and drive phases. However, despite the high optimization performance, the algorithm significantly increased computation time.

The algorithm incorporates five key parameters that require calibration: NIS, NSV, GRSV, GRCV and MNR. Proper calibration of these parameters is crucial, particularly for MNR and NIS, which have the greatest impact on the resulting objective function.

Visualization of the optimized designs confirmed the compatibility of the Z1 and Z3 results with the DoE findings, while the variability in Z2 highlighted the complexity of its optimization. In addition, the results illustrated in Figure 17 show a significant improvement in the reproduction of ground reaction force curves during the heel-strike and propulsion phases. Characteristic oscillations due to the thin shell material were observed during heel-strike contact.

The oscillations observed in the simulated vertical ground reaction force are particularly noticeable during the initial stance phase—heel strike. Similar oscillatory patterns have been reported in another studies [57,58]. These oscillations are mainly due to the heel contact occurring at this stage of the gait cycle.

The considerable kinetic energy produced during heel strike appears challenging to absorb and dissipate instantly, unlike the behavior exhibited by real human feet. Previous studies have shown that a considerable part of these oscillations may be due to numerical effects related to dynamic integration. This phenomenon can be attributed to the contact algorithm used in simulation software to manage contact mechanics [58].

Although the study focused on optimizing prosthesis performance, potential limitations of the FEM were considered. The applied boundary conditions were validated against experimental data obtained from motion capture and force plate measurements to ensure model reliability. Material properties were selected based on literature data commonly used in similar simulations. However, it is acknowledged that certain simplifications inherent in the gait model and assumptions regarding material properties may affect the accuracy of the simulation. Future research could explore refining the contact model and incorporating more advanced representations of material behavior to further enhance the simulation’s predictive accuracy.

Analyzing the results of optimizing the multi-material topology, it was observed that when the angle between the applied force and the constraint region was about 90 degrees, both auxetic and conventional materials were used in the fixation area. This approach optimized the fixation region in terms of energy dissipation and stability. Moreover, a comparison of the three analyzed cases showed that the use of an auxetic material in the internal structure, between the constraint and the load areas, is beneficial for efficient energy dissipation. This strategy could reduce stress concentration in critical areas of the structure, increase its resistance to damage and improve the overall stability of the system under dynamic loads.

The multi-material topology optimization conducted for a wide range of Poisson’s ratio values demonstrated that the combined use of auxetic and conventional materials in the fixation area is advantageous, provided their properties are appropriately tailored.

Regarding the scalability of auxetic materials for mass production, current manufacturing methods, such as advanced additive manufacturing, allow for producing auxetic structures on a small scale. However, large-scale production may face limitations due to material costs and processing times. Future research could investigate industrial-scale fabrication methods to ensure the practical viability of auxetic materials in commercial prosthetic designs.

## 5. Conclusions

The algorithms presented in this study enable a detailed analysis of the influence of both geometric and material parameters on the mechanical and biomechanical characteristics of a prosthetic foot. The applied optimization algorithms provide tools for improving the design of these structures. However, an important aspect that must be considered is the optimization of computation time, which is a critical element in the process of designing and implementing innovative solutions.

This study confirms the hypothesis that it is possible, through the application of an appropriate optimization algorithm, to adapt the prosthetic foot’s response to match the physiological limb in terms of the recorded vertical ground reaction force profile. The optimization algorithm allowed for the precise selection of geometric parameters that adjust the behavior of the designed prosthetic foot to the performance of a natural, physiological limb.

The developed optimization algorithm offers adaptability by allowing for the definition of patient-specific boundary conditions, enabling customization for various patient populations. Additionally, the flexibility of the generated prosthesis geometry enables modifications to the initial design, facilitating the application of the optimization framework to diverse prosthetic configurations and individualized biomechanical requirements.

## Figures and Tables

**Table 1:** The properties of the applied model of the hyperelastic material.

Model	Young’s Modulus [MPa]	Poisson’s Ratio [-]
Hyperelastic (Neo-Hookean)	10	0.35

**Table 2:** Ranges of the values of the analyzed geometrical parameters of the prosthesis.

Parameter	Minimum Value [mm]	Maximum Value [mm]
Z1	2	19
Z2	1	20
Z3	−2	30

**Table 3:** Ranges of values for the tested parameters of the VOA in prosthesis optimization.

Parameter	Minimum Value	Maximum Value
MNR	3	25
NIS	1	5
NSV	1	5
GRSV	1	5
GRCV	1	5

**Table 4:** Properties of the materials applied [28,52].

Material	Young’s Modulus [MPa]	Poisson’s Ratio [-]
Onyx	1400	0.330
Nylon 12	2310	0.408
Steel	230,000	0.200

**Table 5:** Poisson’s ratio values with the corresponding material number.

Material Number	Poisson’s Ratio [-]
0	—
1	−0.5
2	−0.4
3	−0.3
4	−0.2
5	−0.1
6	0.0
7	0.1
8	0.2
9	0.3
10	0.4
11	0.5

**Table 6:** Comparison of parameters obtained for the best solutions.

Parameter	Objective Function Value [-]	Z1 [mm]	Z2 [mm]	Z3 [mm]
MNR = 25	0.0057	1.00	15.31	30.00
NIS = 5	0.0055	1.31	3.57	30.00

**Table 7:** Comparison of computation times for different parameters of the VOA.

Parameter	Value [-]	Objective Function Value [-]	Time [s]
MNR_min_	3	0.0469	8623
MNR_max_	25	0.0057	34,134
NIS_min_	1	0.0295	12,180
NIS_max_	5	0.0055	82,151

## Data Availability

The original contributions presented in this study are included in the article. Further inquiries can be directed to the corresponding author.

## References

[1] Stark G. (2005). Perspectives on How and Why Feet Are Prescribed. JPO J. Prosthet. Orthot..

[2] Koehler-McNicholas S.R., Slater B.C.S., Koester K., Nickel E.A., Ferguson J.E., Hansen A.H. (2018). Bimodal Ankle-Foot Prosthesis for Enhanced Standing Stability. PLoS ONE.

[3] Koehler-McNicholas S.R., Nickel E.A., Barrons K., Blaharski K.E., Dellamano C.A., Ray S.F., Schnall B.L., Hendershot B.D., Hansen A.H. (2018). Mechanical and Dynamic Characterization of Prosthetic Feet for High Activity Users During Weighted and Unweighted Walking. PLoS ONE.

[4] Zelik K.E., Collins S.H., Adamczyk P.G., Segal A.D., Klute G.K., Morgenroth D.C., Hahn M.E., Orendurff M.S., Czerniecki J.M., Kuo A.D. (2011). Systematic Variation of Prosthetic Foot Spring Affects Center-of-Mass Mechanics and Metabolic Cost During Walking. IEEE Trans. Neural Syst. Rehabil. Eng..

[5] Halsne E.G., Czerniecki J.M., Shofer J.B., Morgenroth D.C. (2020). The Effect of Prosthetic Foot Stiffness on Foot-Ankle Biomechanics and Relative Foot Stiffness Perception in People with Transtibial Amputation. Clin. Biomech..

[6] Glanzer E.M., Adamczyk P.G. (2018). Design and Validation of a Semi-Active Variable Stiffness Foot Prosthesis. IEEE Trans. Neural Syst. Rehabil. Eng..

[7] Shell C.E., Segal A.D., Klute G.K., Neptune R.R. (2017). The Effects of Prosthetic Foot Stiffness on Transtibial Amputee Walking Mechanics and Balance Control During Turning. Clin. Biomech..

[8] Adamczyk P.G., Roland M., Hahn M.E. (2017). Sensitivity of Biomechanical Outcomes to Independent Variations of Hindfoot and Forefoot Stiffness in Foot Prostheses. Hum. Mov. Sci..

[9] Major M.J., Twiste M., Kenney L.P.J., Howard D. (2014). The Effects of Prosthetic Ankle Stiffness on Ankle and Knee Kinematics, Prosthetic Limb Loading, and Net Metabolic Cost of Trans-Tibial Amputee Gait. Clin. Biomech..

[10] Fey N.P., Klute G.K., Neptune R.R. (2011). The Influence of Energy Storage and Return Foot Stiffness on Walking Mechanics and Muscle Activity in Below-Knee Amputees. Clin. Biomech..

[11] Ventura J.D., Klute G.K., Neptune R.R. (2011). The Effect of Prosthetic Ankle Energy Storage and Return Properties on Muscle Activity in Below-Knee Amputee Walking. Gait Posture.

[12] Klodd E., Hansen A., Fatone S., Edwards M. (2010). Effects of Prosthetic Foot Forefoot Flexibility on Gait of Unilateral Transtibial Prosthesis Users. J. Rehabil. Res. Dev..

[13] Ármannsdóttir A.L., Lecomte C., Brynjólfsson S., Briem K. (2021). Task Dependent Changes in Mechanical and Biomechanical Measures Result from Manipulating Stiffness Settings in a Prosthetic Foot. Clin. Biomech..

[14] Shepherd M.K., Rouse E.J. (2017). The VSPA Foot: A Quasi-Passive Ankle-Foot Prosthesis with Continuously Variable Stiffness. IEEE Trans. Neural Syst. Rehabil. Eng..

[15] Chauhan P., Singh A.K., Raghuwanshi N.K. (2022). The State of Art Review on Prosthetic Feet and Its Significance to Imitate the Biomechanics of Human Ankle-Foot. Materials Today: Proceedings.

[16] Robbins S., Waked E., Krouglicof N. (2001). Vertical Impact Increase in Middle Age May Explain Idiopathic Weight-Bearing Joint Osteoarthritis. Arch. Phys. Med. Rehabil..

[17] Hansen A.H., Childress D.S. (2005). Effects of Adding Weight to the Torso on Roll-Over Characteristics of Walking. J. Rehabil. Res. Dev..

[18] Olesnavage K.M., Prost V., Johnson W.B., Winter A.G. (2018). Passive Prosthetic Foot Shape and Size Optimization Using Lower Leg Trajectory Error. J. Mech. Des..

[19] Olesnavage K., Winter A. Lower Leg Trajectory Error: A Novel Optimization Parameter for Designing Passive Prosthetic Feet. Proceedings of the 2015 IEEE International Conference on Rehabilitation Robotics (ICORR).

[20] Prost V., Johnson W.B., Kent J.A., Major M.J., Winter A.G. (2022). Biomechanical Evaluation Over Level Ground Walking of User-Specific Prosthetic Feet Designed Using the Lower Leg Trajectory Error Framework. Sci. Rep..

[21] Prost V., Peterson H.V., Winter A.G. (2023). Multi-Keel Passive Prosthetic Foot Design Optimization Using the Lower Leg Trajectory Error Framework. J. Mech. Robot..

[22] Fey N.P., Klute G.K., Neptune R.R. (2012). Optimization of Prosthetic Foot Stiffness to Reduce Metabolic Cost and Intact Knee Loading During Below-Knee Amputee Walking: A Theoretical Study. J. Biomech. Eng..

[23] Rosinha I.P., Gernaey K.V., Woodley J.M., Krühne U. (2015). Topology Optimization for Biocatalytic Microreactor Configurations. Computers and Mathematics with Applications.

[24] Chiriac O.A., Bucur D. (2020). From Conventional Prosthetic Feet to Bionic Feet: A Review. Proceedings of the International Conference of Mechatronics and Cyber-MixMechatronics—2020.

[25] Rajput S., Burde H., Singh U.S., Kajaria H., Bhagchandani R.K. (2021). Optimization of Prosthetic Leg Using Generative Design and Compliant Mechanism. Mater. Today Proc..

[26] Ramalingam B., Srikanth S.A., Bharanidaran R. (2017). Design of a Compliant Mechanism Based Prosthetic Foot. https://www.researchgate.net/publication/317749382_Design_of_a_compliant_mechanism_based_prosthetic_foot.

[27] Fey N.P., Seepersad C.C., Neptune R.R. (2009). Topology Optimization and Freeform Fabrication Framework for Developing Prosthetic Feet. https://www.researchgate.net/publication/261706983_Topology_Optimization_and_Freeform_Fabrication_Framework_for_Developing_Prosthetic_Feet.

[28] Chaari F., Gherardini F., Cavas-Martínez F., Haddar M., Kwon Y., Trojanowska J., Xu J. Lecture Notes in Mechanical Engineering Series Editors. https://link.springer.com/bookseries/11693.

[29] Liang Y.-C., Cuevas Juarez J.R. (2016). A Novel Metaheuristic for Continuous Optimization Problems: Virus Optimization Algorithm. Eng. Optim..

[30] Omenzetter P., Turnbull H., Shull P.J. (2018). Comparison of Two Optimization Algorithms for Fuzzy Finite Element Model Updating for Damage Detection in a Wind Turbine Blade. Nondestructive Characterization and Monitoring of Advanced Materials, Aerospace, Civil Infrastructure, and Transportation XII.

[31] Behnood A., Mohammadi Golafshani E., Hosseini S.M. (2020). Determinants of the Infection Rate of the COVID-19 in the U.S. Using ANFIS and Virus Optimization Algorithm (VOA). Chaos Solitons Fractals.

[32] Aungkulanon P., Luangpaiboon P. (2018). Evolutionary Computation Role in Improving an Accuracy of Forecasting Mortality Data. Int. J. Adv. Soft Comput. Appl..

[33] Grabski J.K., Mrozek A. (2021). Identification of Elastoplastic Properties of Rods from Torsion Test Using Meshless Methods and a Metaheuristic. Comput. Math. Appl..

[34] Grabski J.K., Sopa M., Mrozek A. (2023). Application of the Path-Repairing Technique and Virus Optimization Algorithm for the Dimensional Synthesis of Four-Bar Mechanisms. Arch. Civ. Mech. Eng..

[35] Lim T.-C. (2020). Mechanics of Metamaterials with Negative Parameters.

[36] Zhang Y., Chen Y., Wang T., Zhu Q., Gu M. (2024). Ultrahigh performance passive radiative cooling by hybrid polar dielectric metasurface thermal emitters. Opto-Electron. Adv..

[37] Li Y., Huang X., Liu S., Liang H., Ling Y., Su Y. (2023). Metasurfaces for near-eye display applications. Opto-Electron Sci..

[38] Chen Z., Cheng S., Zhang H., Yi Z., Tang B., Chen J., Zhang J., Tang C. (2024). Ultra wideband absorption absorber based on Dirac semimetallic and graphene metamaterials. Phys. Lett. A.

[39] Lim T.-C. (2015). Auxetic Materials and Structures.

[40] Mrozek A., Strek T. (2022). Numerical Analysis of Dynamic Properties of an Auxetic Structure with Rotating Squares with Holes. Materials.

[41] Duncan O., Shepherd T., Moroney C., Foster L., Venkatraman P.D., Winwood K., Allen T., Alderson A. (2018). Review of Auxetic Materials for Sports Applications: Expanding Options in Comfort and Protection. Appl. Sci..

[42] Bilski M., Wojciechowski K.W., Stręk T., Kędziora P., Grima-Cornish J.N., Dudek M.R. (2021). Extremely Non-Auxetic Behavior of a Typical Auxetic Microstructure Due to Its Material Properties. Materials.

[43] Krishnan B.R., Biswas A.N., Kumar K.V.A., Sreekanth P.S.R. (2022). Auxetic Structure Metamaterial for Crash Safety of Sports Helmet. Mater. Today Proc..

[44] Tahir D., Zhang M., Hu H. (2022). Auxetic Materials for Personal Protection: A Review. Phys. Status Solidi (B).

[45] Fardan M.F., Lenggana B.W., Ubaidillah U., Choi S.B., Susilo D.D., Khan S.Z. (2023). Revolutionizing Prosthetic Design with Auxetic Metamaterials and Structures: A Review of Mechanical Properties and Limitations. Micromachines.

[46] Devin K.M., Tang J., Hamilton A.R., Moser D., Jiang L. (2024). Assessment of 3D Printed Mechanical Metamaterials for Prosthetic Liners. Proc. Inst. Mech. Eng. Part H J. Eng. Med..

[47] Brown N., Owen M.K., DesJardins J.D., Garland A., Fadel G.M. (2020). Metamaterial Design for Targeted Limb-Socket Interface Pressure Offloading in Transtibial Amputees. Proceedings of the ASME Design Engineering Technical Conference.

[48] Kowalczyk M., Jopek H. (2020). Numerical Analysis of the Lower Limb Prosthesis Subjected to Various Load Conditions. Vib. Phys. Syst..

[49] Um H.J., Kim H.S., Hong W., Kim H.S., Hur P. (2021). Design of 3D Printable Prosthetic Foot to Implement Nonlinear Stiffness Behavior of Human Toe Joint Based on Finite Element Analysis. Sci. Rep..

[50] Kim H.S., Um H.J., Hong W., Kim H.S., Hur P. Structural Design for Energy Absorption During Heel Strike Using the Auxetic Structure in the Heel Part of the Prosthetic Foot. Proceedings of the 2021 18th International Conference on Ubiquitous Robots (UR).

[51] Ewins D., Collins T. (2014). Clinical Gait Analysis. Clinical Engineering.

[52] Winter D.A. (2009). Biomechanics and Motor Control of Human Movement.

[53] FEBio. https://febio.org/.

[54] Andreassen E., Clausen A., Schevenels M., Lazarov B.S., Sigmund O. (2011). Efficient Topology Optimization in MATLAB Using 88 Lines of Code. Struct. Multidiscip. Optim..

[55] Zheng R., Yi B., Peng X., Yoon G.H. (2024). An Efficient Code for the Multi-Material Topology Optimization of 2D/3D Continuum Structures Written in Matlab. Appl. Sci..

[56] Siddiqui M.I.H., Alnaser I.A., Alluhydan K. (2023). Assessment of a Carbon Fiber Prosthetic Running Blade for Enhanced Reliability. Eksploat. I Niezawodn..

[57] Dai X.Q., Li Y., Zhang M., Cheung J.T. (2006). Effect of Sock on Biomechanical Responses of Foot during Walking. Clin. Biomech..

[58] Qian Z., Ren L., Ding Y., Hutchinson J.R., Ren L. (2013). A dynamic finite element analysis of human foot complex in the sagittal plane during level walking. PLoS ONE.

